# The Impact of Perceived Algorithmic Control on Gig Workers’ Turnover Intention: Mediating Roles of Perceived Usefulness and Psychological Contract Breach

**DOI:** 10.3390/bs16050764

**Published:** 2026-05-13

**Authors:** Anbo Wu, Shihao Zhang, Linhui Sun, Xiaofang Yuan, Xinping Wang

**Affiliations:** 1 School of Management, Xi’an University of Science and Technology, Xi’an 710054, China; anbowu@xust.edu.cn (A.W.); 24202230112@stu.xust.edu.cn (S.Z.); linhuisun@xust.edu.cn (L.S.); yxf@xust.edu.cn (X.Y.); 2 Energy Economic Research Center, Xi’an University of Science and Technology, Xi’an 710054, China

**Keywords:** perceived algorithmic control, psychological contract breach, perceived usefulness, turnover intention, time pressure

## Abstract

In recent years, digital technologies have been widely applied in the gig economy, and platform algorithms have become the core tool for managing gig workers. This study collected data through a multi-phase questionnaire survey and conducted empirical regression tests and fuzzy-set Qualitative Comparative Analysis (fsQCA). It explored how perceived algorithmic control affected gig workers’ turnover intention through psychological contract breach and perceived usefulness, analyzed the moderating role of time pressure, and investigated its relevant configurational pathways. The results indicated that perceived algorithmic control negatively affected gig workers’ turnover intention. Psychological contract breach and perceived usefulness both partially mediated the relationship between perceived algorithmic control and turnover intention. Time pressure negatively moderated the relationship between perceived algorithmic control and perceived usefulness, thereby weakening the indirect effect of perceived algorithmic control on turnover intention via perceived usefulness; however, it did not significantly moderate the relationship between perceived algorithmic control and psychological contract breach. Configurational analysis revealed that high psychological contract breach served as a core condition leading to turnover intention, and its combination with low perceived usefulness or low perceived algorithmic control constituted multiple pathways toward high turnover intention. This study extends the literature on the behavioral consequences of perceived algorithmic control and provides theoretical insights and practical implications for gig platforms seeking to optimize algorithm design, alleviate time pressure, and reduce worker turnover.

## 1. Introduction

In recent years, the gig economy, as a new economic model centered on flexible employment, has developed rapidly across the world. As technology continues to reshape the labor market, China has become a leading country in the global gig economy. By 2024, the number of flexible employment workers in China had reached 265 million, of which 175 million were platform-based gig workers. Against this background, the algorithm system behind digital platforms is no longer merely a technical tool. It has evolved into a core institutional carrier for platform enterprises to restructure labor relations and implement labor governance, and has become a new form of management actor that profoundly reshapes traditional employment relations and human resource management practices. With flexible and independent work arrangements (Duggan et al., 2020), this new employment model enables efficient matching of labor supply and demand through algorithms (Wood et al., 2019). It not only creates huge economic value but also gives rise to a series of practical issues, such as labor process control and labor rights protection, providing a new perspective for the study of employment relations and labor behavior in the gig economy (Kadolkar et al., 2025).

However, with the rapid expansion of the gig economy, gig workers are also facing practical dilemmas of high mobility and occupational uncertainty. In this context, identifying the drivers of gig workers’ turnover, reducing turnover through algorithmic management, and understanding how algorithms function in the gig economy have become core issues in organizational behavior and human resource management research (S. Yu et al., 2025). It is particularly worth noting that in the gig economy, a typical unstable labor market, low job switching costs, general lack of social security and individual structural dependence on platforms mean that retention is not necessarily equivalent to job satisfaction or well-being improvement, and turnover is not always a negative behavioral outcome (Dantas, 2019). Therefore, when exploring the impact of perceived algorithmic control on turnover intention, this study aims to reveal the underlying psychological and cognitive mechanisms, rather than presupposing the inhibition of turnover intention as a self-evident positive management goal.

In the unique labor context of the gig economy, algorithms, as a key technological intermediary, connect gig workers, platform enterprises, and service interfaces, and run through the entire labor service process (Duggan et al., 2020). From an objective perspective, algorithmic control functions as a systematic governance mechanism through which platform enterprises structure labor processes, assign tasks, and enforce labor discipline. It enables fine-grained control over the entire labor process through standardized procedures and essentially serves as a core tool for platforms to obtain labor value (Stark & Pais, 2021). From the perspective of subjective experience, perceived algorithmic control is the comprehensive perception, evaluation and behavioral response of gig workers to this objective control mechanism. The two are related to each other but also have clear conceptual boundaries. In this process, algorithmic control exhibits a dual attribute of bidirectional interaction. On the one hand, it reflects the active control exerted by platform algorithms. Platform algorithmic control improves labor efficiency, enhances workers’ self-efficacy and willingness to continue participation through material incentives and gamified design (Duggan et al., 2023), but its standardized management model may also intensify work pressure and induce deviant behaviors (C. Wang et al., 2025; Zhang et al., 2023). On the other hand, gig workers do not passively accept algorithmic control. Instead, they exert feedback effects on algorithmic systems through strategic avoidance, rule gaming, behavioral manipulation and other methods (Kadolkar et al., 2025). For example, they deliberately adjust order receiving strategies, use algorithm loopholes to optimize performance, and carry out collective negotiations to resist unreasonable algorithm rules, in an attempt to influence algorithms. This dynamic interaction between platform algorithmic control and workers’ responses, combined with the high autonomy and mobility of gig work, makes the formation mechanism of turnover intention more complex than in traditional employment relationships (P. M. Lin et al., 2020). However, existing studies have not fully explored the deep psychological and cognitive mechanisms of how perceived algorithmic control affects gig workers’ turnover intention.

Against this backdrop, psychological contract theory provides a key perspective for revealing the underlying mechanisms. A psychological contract refers to an implicit contractual relationship reached between an organization and its employees based on expectations, trust, and commitments (Argyris, 1960). In the context of traditional employment models, psychological contract breach has been widely shown to be an important mediating factor leading to employees’ turnover intention. According to the Conservation of Resources (COR) theory, individuals tend to preserve their resources and avoid resource loss (Hobfoll, 1989). When gig workers perceive that the platform’s algorithmic system fulfills its commitments and maintains a balance between resource input and return, the psychological contract may be strengthened, thereby reducing the risk of psychological contract breach and ultimately lowering turnover intention. On the other hand, perceived usefulness, as a core variable in the technology acceptance model, reflects an individual’s evaluation of whether using a certain technical tool can improve their work performance (Davis, 1989). In the context of gig work, workers’ perceived usefulness of the algorithmic control system directly affects their acceptance of algorithm tools and job satisfaction. If workers believe that the information provided by the algorithm system can effectively support their work, they are more likely to develop a positive work attitude, thus suppressing turnover intention to a certain extent (Berger et al., 2019). Therefore, this study introduces psychological contract breach and perceived usefulness as mediating variables into the model to explore the internal psychological and cognitive mechanisms through which perceived algorithmic control influences gig workers’ turnover intention.

In addition, gig workers inevitably experience time constraints in the course of their work. For example, short task completion deadlines, parallel multi-tasking, customer order reminders and uncertain factors jointly constitute the sources of time pressure. Relevant studies have shown that time pressure is one of the important factors leading to work stress and occupational burnout among workers (Gao et al., 2023). Meanwhile, considering that individuals vary in their perception and tolerance of time pressure, such differences may further moderate the action path between perceived algorithmic control and turnover intention. Therefore, this paper introduces time pressure as a moderating variable into the research framework to explore its boundary effects across different causal pathways.

In summary, this study adopts the Conservation of Resources Theory as the core theoretical perspective because it provides a higher-level framework that integrates core concerns under different theoretical traditions into the basic concept of resource conservation and acquisition. Meanwhile, the technology acceptance model and psychological contract theory are incorporated as complementary transmission mechanisms within the framework of conservation of resources theory. Within this framework, focusing on the Chinese gig economy context, this study constructs a dual mediation model in which perceived algorithmic control influences turnover intention through psychological contract breach and perceived usefulness, and examines the boundary effects of these resource transmission paths under different levels of time pressure. In addition, combined with fsQCA configurational analysis, this study further explores the combined effects of multiple resource conditions on turnover intention. It provides theoretical and practical references for gig platforms to optimize algorithm design, reduce workers’ resource loss and lower mobility.

## 2. Literature Review

This section systematically reviews and synthesizes the relevant literature concerning the impact of perceived algorithmic control on gig workers’ turnover intention, thereby clarifying the theoretical linkages among the focal constructs.

### 2.1. Perceived Algorithmic Control and Labor Management Practices in the Gig Economy

Perceived algorithmic control refers to workers’ overall perception of the dynamic control mechanisms implemented by platform algorithms in the labor process, including three dimensions: normative guidance, tracking and evaluation, and behavioral constraints (Pei et al., 2021). It should be emphasized that algorithmic control is fundamentally an objective organizational and governance mechanism that continuously regulates the labor process through data-driven rules. In contrast, perceived algorithmic control reflects workers’ subjective evaluations of this mechanism in terms of efficiency, fairness, and support. Therefore, this concept is not a substitute for control itself, but a depiction of workers’ psychological responses to it.

Existing studies show that perceived algorithmic control has a double-edged sword effect on gig workers. On the one hand, the management model under algorithmic control, while improving operational efficiency, has intensified the information monopoly of platforms and strengthened workers’ dependent relationship with platforms (Q. Lin et al., 2025; Wood et al., 2019). On the other hand, platform algorithmic control endows gig workers with flexibility and autonomy, yet it also brings negative impacts such as social isolation and overwork (Lee, 2018). These complex effects indicate that perceived algorithmic control, as a contextual factor unique to the gig economy, profoundly influences gig workers’ work experience and psychological states. Accordingly, its relationship with turnover intention warrants more systematic and theory-driven investigation.

### 2.2. Psychological Mechanisms and Technology Perception Paths of Turnover Intention

In the gig work context, low job switching costs make the formation of turnover intention more complex, which has become a key issue constraining the sustainable development of the gig economy. In early research, Mobley’s turnover decision model posited that turnover decisions result from a cognitive evaluation process involving job satisfaction, perceived alternative employment opportunities, and the expected utility of such alternatives (Mobley, 1977). Subsequent studies have further confirmed that turnover intention is the direct antecedent variable of turnover behavior, and actual turnover is also moderated by turnover costs and external opportunities (Miller et al., 1979).

In the research on the influence mechanism of turnover intention, psychological contract breach and perceived usefulness are two key variables. Psychological contract was first proposed by Argyris, describing the implicit and unstated mutual expectations between employees and organizations (Argyris, 1960). Traditionally, psychological contract breach refers to employees’ cognitive evaluation of an organization’s failure to fulfill its implicit promises (Robinson & Wolfe Morrison, 2000). However, in the gig economy context, platforms do not recognize standard employment relationships, rights and obligations are highly ambiguous, and exchange relationships are asymmetric. In this situation, such prerequisite issues as whether a contract exists between workers and platforms, who the other party of the contract is, and what obligations platforms should fulfill have become complex and vague (J. Wang & Tomassetti, 2024). Therefore, in the gig economy, psychological contract breach cannot be simply regarded as an extension of the psychological contract in traditional employment relationships. It is a contextual moral evaluation formed based on algorithm rules, platform rhetoric and vague promises, reflecting workers’ subjective judgment on whether platform algorithms fulfill their commitments. When workers perceive a gap between platform algorithm practices and their implicit promises, psychological contract breach is likely to be triggered (Tomprou & Lee, 2022).

Perceived usefulness, as a core variable of the Technology Acceptance Model (TAM), refers to an individual’s perceptual evaluation of the degree to which using a specific technology or tool improves their work performance (Davis, 1989). Studies have shown that a higher level of perceived usefulness significantly boosts users’ satisfaction with the use of algorithm models (Hassan et al., 2025). However, it is important to note that the theoretical meaning of perceived usefulness needs to be carefully defined in the context of platform algorithm management. Perceived usefulness in the traditional TAM framework is used to predict users’ voluntary adoption intention of technology, while gig workers do not voluntarily choose to use platform algorithms. Algorithm rules are usually mandatory, and non-acceptance means exiting the platform. As revealed by research, algorithm management is essentially an algorithmic discipline mechanism whose operation does not depend on workers’ subjective identification (J. Wang & Tomassetti, 2024). Therefore, this study defines perceived usefulness as workers’ evaluation of the extent to which the algorithm system effectively supports task completion, rather than adopting the predictive logic of technology adoption and usage intention in the traditional TAM framework.

### 2.3. The Situational Moderating Role of Time Pressure

Time pressure generally refers to a kind of stress perception that individuals experience due to the scarcity of time resources in the work process (Tjahjadi & Cahyadi, 2020). In the gig context, time pressure does not only stem from the urgency of tasks themselves, but is deeply embedded in the operational logic of the platform’s algorithmic control system. Specifically, platforms institutionalize and normalize time constraints through dynamic scheduling mechanisms, such as delivery time limits and countdown reminders. These mechanisms function as key operational tools embedded in the algorithmic control system, enabling continuous guidance and constraint of workers’ behavior. At the same time, external environmental factors, such as customer reminders and parallel processing of multiple tasks, further superimpose and strengthen individuals’ experience of time pressure. Therefore, in the gig labor process, time pressure is not only a situational external source of pressure, but also an important functional carrier relied on by the platform algorithmic control system in its operation. The two intertwine with each other and jointly shape workers’ perception of time pressure. In the gig work context, time pressure has been proven to be a key factor causing work stress among workers (Gao et al., 2023). Therefore, this study further explores its boundary condition in the impact of perceived algorithmic control on turnover intention.

### 2.4. Literature Review and Summary

In summary, existing studies have laid a foundation for understanding the behavior of gig workers, but still have the following shortcomings. Most existing studies discuss the functional relationships among variables in isolation, and fail to conduct an integrated analysis of Conservation of Resources Theory, Psychological Contract Theory and Technology Acceptance Model. They do not systematically explain the internal mechanism through which perceived algorithmic control affects turnover intention, and their theoretical perspectives are relatively single, making it difficult to explain how algorithmic control influences turnover intention through different resource mechanisms. Research on the regulatory boundary of time pressure is not in-depth enough, and it is not clearly explained how time pressure regulates the relationships between perceived algorithmic control and different variables. Therefore, this paper aims to construct a moderated dual mediation model by integrating Psychological Contract Theory, Technology Acceptance Model and Conservation of Resources Theory, so as to reveal the impact of perceived algorithmic control on turnover intention and its internal mechanism, and make up for the deficiencies of a single research perspective. Second, this study clarifies the contextual regulatory role of time pressure and refines the boundary conditions under which perceived algorithmic control affects turnover intention. Meanwhile, this study extends Psychological Contract Theory from traditional organizational relationships to the gig economy context, expanding the applicability and explanatory power of relevant theories in the gig economy scenario.

## 3. Theoretical Foundation and Research Hypotheses

### 3.1. Perceived Algorithmic Control and Turnover Intention

In the digital platform context, algorithms conduct real-time intervention and regulation on the labor process through embedded rule systems and data logic. Labor process theory emphasizes that technology is not merely a production tool, but also an important means for enterprises to realize management control and regulate the labor process. The platform governance perspective further suggests that, in addition to optimizing efficiency, algorithms serve as core mechanisms through which platform organizations define labor relations, allocate labor resources, and implement disciplinary constraints. Thus, algorithm management is not a technical tool that only needs to be passively accepted, but an institutional arrangement deeply embedded in power relations (J. Wang & Tomassetti, 2024). However, macro-level institutional arrangements ultimately influence individual behavior through subjective perception and psychological processing (Yuan et al., 2025; Yuan et al., 2026). Based on this premise, the study examines the cognitive logic and psychological responses at the individual level by drawing on the Conservation of Resources Theory and the Technology Acceptance Model.

Focusing on the gig economy context, algorithms provide multiple safeguards for workers to reduce their own resource loss in different dimensions (Kellogg et al., 2020). First, in terms of normative guidance, platform algorithm systems can achieve accurate order matching and information push by comprehensively considering multiple factors such as workers’ locations, working abilities and merchants’ order demands based on big data and artificial intelligence technologies. This intelligent allocation mechanism reduces workers’ search costs and decision burdens caused by information asymmetry and passive order-taking. It also mitigates the loss of cognitive and emotional resources, thereby providing an important basis for workers to remain on the platform. Second, in terms of tracking and evaluation, algorithm systems can feed back workers’ work progress in real time and provide timely information support, such as abnormal reminders and route optimization (Deng et al., 2025). It enhances workers’ sense of control and predictability over the labor process, thereby reducing anxiety and psychological resource loss caused by uncertainty (S. Yu et al., 2025). Finally, in terms of behavioral constraint, algorithm systems can provide workers with additional economic returns and psychological incentives through mechanisms such as order peak subsidies, bad weather price premiums and gamified achievement rewards (Wee & Choong, 2019). This directly corresponds to the motivation of individuals to strive for material resources and personal achievement in the Conservation of Resources Theory. Algorithms make the link between effort and reward more transparent, immediate, and predictable, thereby strengthening workers’ perceptions of input–output fairness. When workers believe that they can steadily obtain and accumulate resources by following algorithm rules, their likelihood of leaving the platform will naturally decrease.

In summary, although algorithmic control embeds structural power and control logic, its function as a key resource provider and protector plays a more central role in shaping workers’ intention to stay at the level of individual subjective perception and turnover decision. The erosion of autonomy and induction of pressure caused by algorithms may more greatly affect immediate work experiences, such as job satisfaction and job burnout. As a long-term exit decision, turnover intention fundamentally depends on workers’ rational or semi-rational judgment on whether the platform can continuously meet their core resource acquisition needs. On this basis, this paper puts forward the following hypotheses:

**H1.**  *Perceived algorithmic control is negatively associated with gig workers’ turnover intention.*

### 3.2. The Mediating Role of Psychological Contract Breach

In the gig economy, although platforms usually refuse to recognize formal employment relations, a reciprocal exchange relationship based on the intermediation of algorithm systems actually still exists between workers and platforms (X. Li et al., 2023). Platforms convey a series of implicit promises to workers through algorithm systems, which form workers’ psychological expectations of platform obligations (Pei et al., 2021; Saksida et al., 2026). Workers provide labor through algorithm systems while expecting platforms to fulfill commitments related to efficiency support, economic returns, and fair treatment. When workers perceive that the algorithm system is consistent with platform promises in rule implementation, task allocation, reward settlement and other links, their evaluation of platform performance tends to be positive, and the risk of psychological contract breach decreases accordingly. At the same time, algorithm-driven reward mechanisms enable workers to clearly and timely convert work performance into predictable economic returns. Although workers receive monetary compensation, the genuine satisfaction they gain from work will strengthen their sense of belonging and identification with the platform, thus consolidating the stability of employment relations and reducing turnover intention (J. Wang & Tomassetti, 2024; J. Yu & Hamid, 2024). In addition, compared with the subjective decision-making biases that may exist in traditional employment relationships, algorithm-driven gig platforms can achieve high levels of procedural and distributive justice through technological governance mechanisms (Lee, 2018). This makes it easier for workers to perceive that the platform algorithm fulfills the promise of fair treatment, further reduces the likelihood of psychological contract breach, strengthens the contractual relationship with the platform, and ultimately reduces the emergence of turnover intention.

Thus, algorithmic control effectively reduces workers’ concerns about the loss of platform relational resources such as trust and sense of fairness by ensuring procedural fairness, information transparency and rule consistency, thus avoiding the relational resource crisis caused by psychological contract breach and ultimately restraining the formation of turnover intention. Based on the above reasoning, the following hypothesis is proposed:

**H2.**  *Psychological contract breach mediates the relationship between perceived algorithmic control and gig workers’ turnover intention.*

### 3.3. The Mediating Role of Perceived Usefulness

Although perceived usefulness is derived from the Technology Acceptance Model, algorithm systems are not neutral technical tools in the gig economy context, but institutional arrangements embedded with the logic of labor control. Therefore, workers’ judgment of “usefulness” not only involves efficiency improvement, but also includes a comprehensive evaluation of the rationality, fairness and acceptability of control. In other words, perceived usefulness in this study not only reflects instrumental value but also represents workers’ assessment of the cognitive legitimacy of the algorithmic control mechanism. When workers perceive that the algorithm system is highly practical, they are more likely to form a positive attitude toward the platform, thereby reducing their turnover intention.

Specifically, platform algorithms align order demand with workers’ real-time geographic locations through data-driven matching mechanisms, substantially reducing idle waiting time; dynamic route optimization minimizes unnecessary detours and time loss, thereby enhancing task execution efficiency (Wu et al., 2023). Although dynamic rating and performance evaluation systems impose a degree of behavioral constraints, they also generate data-driven feedback that facilitates service improvement and performance enhancement in gig work contexts. These algorithmic functions directly meet gig workers’ core needs for improving work efficiency, increasing income and receiving fair feedback (W. Li et al., 2024), enabling gig workers to more clearly perceive the instrumental value of algorithmic technology for enhancing work performance in task allocation, process management and performance evaluation. When workers perceive that algorithmic control can effectively improve their work efficiency and reduce ineffective labor and resource waste, their evaluation of algorithmic management may shift from perceiving it as external surveillance to viewing it as performance-enabling governance, thereby strengthening the perceived usefulness of the algorithmic system (Davis, 1989). Such positive evaluations further reinforce job satisfaction, alleviate work-related strain and psychological fatigue, and increase workers’ acceptance of existing work arrangements and perceived platform value. As a result, workers may exhibit stronger intentions to remain on the platform, thereby diminishing turnover intention (Jabagi et al., 2025).

From the perspective of Conservation of Resources Theory, perceived algorithmic control enhances perceived usefulness because it provides workers with valuable instrumental resources. To continuously obtain such resource gains, workers will be more inclined to stay on the current platform, and their turnover intention will naturally decrease accordingly. Based on this reasoning, the following hypothesis is proposed:

**H3.**  *Perceived usefulness mediates the relationship between perceived algorithmic control and gig workers’ turnover intention.*

### 3.4. The Moderating Role of Time Pressure

In the gig work context, time pressure is almost ubiquitous and serves as a key contextual factor influencing workers’ behaviors and psychological states (Lascău et al., 2024). This section will, respectively, discuss the moderating role of time pressure in the two different paths.

First, time pressure moderates the relationship between perceived algorithmic control and psychological contract breach by changing workers’ cognitive judgments of platform performance. When workers are under high time pressure, they are forced to invest more physical and psychological resources to avoid overtime fines and complete task indicators, and even take risky behaviors to ensure task progress (Hsu et al., 2024). Long-term overwork is likely to cause emotional exhaustion and job burnout (Chen et al., 2022; X. Wang et al., 2026). At this time, the platform algorithm system is more likely to be perceived as a tool of exploitation rather than assistance. Such negative attribution will strengthen workers’ sense of distrust, making them believe that the platform has failed to fulfill its implicit promises of reasonable task allocation and fair treatment (Semujanga & Parent-Rocheleau, 2024). Continuous resource consumption deepens the degree of psychological contract breach between workers and the platform. Conversely, in a low time pressure context, workers have sufficient cognitive resources to understand the operation logic of algorithm rules, and are more likely to form rational and positive attribution judgments (Schilbach et al., 2023). This helps alleviate the sense of uncertainty and unfairness caused by algorithmic control and reduces the risk of psychological contract breach. On this basis, the following hypothesis is proposed:

**H4.**  *Time pressure moderates the relationship between perceived algorithmic control and psychological contract breach. That is, the higher the time pressure, the weaker the inhibitory effect of perceived algorithmic control on workers’ psychological contract breach.*

Second, time pressure also moderates the relationship between perceived algorithmic control and perceived usefulness by influencing workers’ cognitive evaluation of the instrumental value of algorithms. When time pressure is high, workers are often in a continuous state of heavy workload. Their cognitive systems tend to be task-oriented and result-oriented, making it difficult for them to devote extra energy to understanding the efficiency advantages of algorithm systems in route optimization, task allocation and other aspects. In this context, algorithmic control is more likely to be regarded as a factor that increases operational complexity or restricts autonomy, thus weakening its perceived value in improving job performance (Wood et al., 2019). This suggests that even if algorithmic control improves efficiency, its positive effect on perceived usefulness may be weakened under high time pressure. In a low time-pressure context, workers have more time and energy to learn and adapt to algorithm systems, and can more clearly experience the practical effects of algorithms in improving work efficiency and reducing uncertainty (Jiang & Sinchaisri, 2025). This enhances their perception of the usefulness of algorithm systems, and such positive cognition further increases their acceptance and willingness to continue using platform algorithms (Liang et al., 2025; Zhang et al., 2026). On this basis, the following hypothesis is proposed:

**H5.**  *Time pressure moderates the relationship between perceived algorithmic control and perceived usefulness. That is, the higher the time pressure, the weaker the promoting effect of perceived algorithmic control on workers’ perceived usefulness.*

### 3.5. The Moderated Mediation Effect

Building upon the preceding hypotheses concerning mediation and moderation, this study further proposes moderated mediation effects. Specifically, time pressure, as a salient situational contingency, may condition the indirect effects of perceived algorithmic control on turnover intention through psychological contract breach and perceived usefulness. Under conditions of high time pressure, the indirect effects transmitted through psychological contract breach and perceived usefulness are expected to be attenuated. Accordingly, the following hypotheses are proposed:

**H6.**  *Time pressure negatively moderates the indirect effect of perceived algorithmic control on turnover intention through psychological contract breach, such that the indirect effect is weaker when time pressure is high.*

**H7.**  *Time pressure negatively moderates the indirect effect of perceived algorithmic control on turnover intention through perceived usefulness, such that the indirect effect is weaker when time pressure is high.*

**Figure 1:**
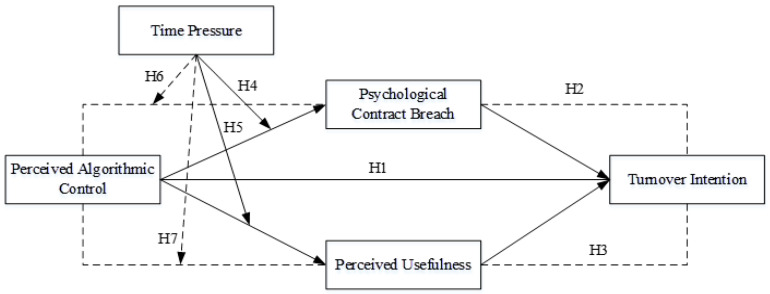
Research Model.

Based on the hypotheses proposed above, the theoretical model of this study is presented in Figure 1.

## 4. Research Methods

To systematically examine how perceived algorithmic control influences gig workers’ turnover intention, this study adopts a phased questionnaire survey and conducts empirical analyses using both regression and configurational methods. The former is used to test the net effects and hypothetical paths among variables, while the latter explores the formation paths of turnover intention under the combined effect of multiple conditions. The overall research design follows a logical sequence of sample selection, variable measurement, and data analysis, ensuring the rigor and coherence of the research process.

### 4.1. Sample Selection and Data Source

This study took food delivery riders, a typical group of gig workers, as the research sample. Participants were recruited on a voluntary basis, and the last four digits of their mobile phone numbers were recorded as questionnaire IDs for subsequent questionnaire matching. The survey was clearly stated to be used only for academic research. Before questionnaire distribution, the purpose of the survey was explained to participants in advance, and the main variables were interpreted to avoid misunderstanding among participants.

To minimize common method bias, the questionnaire was distributed in two stages with an interval of one to two weeks between each stage. In the first round of questionnaire distribution, data were mainly collected on perceived algorithmic control, perceived usefulness, time pressure, and control variables such as gender and age. A total of 240 questionnaires were distributed, and 212 valid questionnaires were obtained after excluding those with obvious logical confusion and routine responses. On this basis, a second round of questionnaires was distributed to mainly collect data on psychological contract breach and turnover intention. A total of 212 questionnaires were distributed in this phase. After excluding responses with incorrect answers to attention-check items or patterned responding, 190 valid questionnaires were obtained. Among the final valid questionnaires, 117 respondents were male workers, accounting for 61.6%; the participants were mainly aged 24 and above, accounting for 82.1% of the total; in terms of educational background, 71.6% held a junior college degree or below; and 135 were full-time workers, accounting for 71.1%. It should be noted that, due to limitations in research resources and geographic scope, all samples are collected from gig workers in China. Therefore, the findings are primarily applicable to the Chinese gig economy context, and caution should be exercised when generalizing them to other countries or institutional settings.

### 4.2. Measurement

This study employs well-established measurement scales from prior research to assess the focal constructs. Some scales are designed for the gig economy scenario and can be directly adapted to this context. For scales derived from English literature, the translation–back-translation procedure is adopted to ensure semantic equivalence and content consistency across languages. Before the formal survey, a pilot test is conducted with a subset of target participants, and item wording is refined based on their feedback to improve clarity and contextual relevance. All scales adopt a 5-point Likert scale, with numbers 1 to 5, respectively, representing “strongly disagree” to “strongly agree”.

Perceived Algorithmic Control: The scale of gig workers’ perceived algorithmic control developed by Pei et al. (2021) was adopted. A representative item is, “The algorithm intelligently allocates my work tasks”. The Cronbach’s α for this scale was 0.827.

Psychological Contract Breach: The scale developed by Robinson and Wolfe Morrison (2000) was adopted. A representative item is, “I believe the platform has successfully fulfilled its commitments to me”. The Cronbach’s α for this scale was 0.875.

Perceived Usefulness: The scale developed by Davis (1989) was adopted. A representative item is, “The platform’s algorithmic system has improved my job performance”. The Cronbach’s α for this scale was 0.763.

Turnover Intention: The scale developed by Scott et al. (1999) was adopted. A representative item is, “Since working on this platform, I have seriously considered leaving it on multiple occasions”. The Cronbach’s α for this scale was 0.878.

Time Pressure: The scale developed by Gao et al. (2023) was adopted. A representative item is, “The system displays and continuously updates the estimated order completion time, creating a sense of urgency”. The Cronbach’s α for this scale was 0.856.

Control Variables: With reference to relevant studies, this study took basic demographic information, including gender, age, educational level and workers’ occupational type as control variables.

### 4.3. Analysis Methods

In the data analysis stage, this study adopts a two-stage analysis strategy. In the first stage, SPSS 27.0 (IBM Corporation, Armonk, NY, USA) is used for hypothesis testing, including descriptive statistics, correlation analysis, hierarchical regression, and tests of mediation and moderation effects. Through this series of analyses, the direct impact of perceived algorithmic control on turnover intention, the mediating roles of psychological contract breach and perceived usefulness, and the moderating role of time pressure are verified. In the second stage, fsQCA 4.1 (Ragin & Davey, Irvine, CA, USA) is employed to identify configurational pathways through which combinations of antecedent conditions lead to turnover intention. This stage includes variable calibration, necessary condition test and conditional configurational analysis. The combination of these two analytical approaches not only retains the explanatory power of linear relationships but also enhances the exploration of multi-factor interactions, providing a more comprehensive understanding of the formation mechanisms underlying gig workers’ turnover intention.

## 5. Research Results

### 5.1. Common Method Bias Test

To control for the potential influence of common method bias on the results, several tests were conducted. First, Harman’s single-factor test was used to examine common method bias. The results showed that, under the unrotated condition, the first factor accounted for 30.96% of the variance, which does not exceed the critical threshold of 40%. Second, the unmeasured latent method construct (ULMC) approach was applied for further testing. In the confirmatory factor analysis, two models were estimated: a five-factor model and a model including a method factor, in which all items load on both their respective constructs and the method factor. By comparing the fit indices of the two models, it was found that the inclusion of the method factor did not lead to a significant improvement in model fit (ΔCFI = 0.018, ΔTLI = 0.016, ΔRMSEA = −0.005, ΔSRMR = −0.009), indicating that there is no serious common method bias in this study.

### 5.2. Confirmatory Factor Analysis

To examine the discriminant validity among the study variables, confirmatory factor analysis (CFA) was conducted for perceived algorithmic control, psychological contract breach, perceived usefulness, time pressure, and turnover intention. The results were presented in Table 1. Compared with other factor models, the five-factor model had a better fit (χ^2^/df = 1.495, CFI = 0.928, TLI = 0.919, RMSEA = 0.051, SRMR = 0.064), which indicated that there was a relatively distinct discriminant validity among all variables.

### 5.3. Descriptive Statistics and Correlation Analysis

Table 2 reports the means, standard deviations, and Pearson correlation coefficients of the study variables. Perceived algorithmic control was significantly negatively correlated with both psychological contract breach (r = −0.425, *p* < 0.01) and turnover intention (r = −0.422, *p* < 0.01), and significantly positively correlated with perceived usefulness (r = 0.547, *p* < 0.01). Psychological contract breach was significantly positively correlated with turnover intention (r = 0.757, *p* < 0.01); perceived usefulness was significantly negatively correlated with turnover intention (r = −0.450, *p* < 0.01). The correlations among the variables were consistent with the research expectations.

### 5.4. Hypothesis Testing

#### 5.4.1. Main Effect Test

This study tested the direct effects and moderating effects through hierarchical regression, with the results of hierarchical regression presented in Table 3. First, control variables including gender, age and educational level were included in Model 9. Perceived algorithmic control was subsequently entered into Model 10 to examine H1. As shown in Model 10, perceived algorithmic control had a significant negative effect on gig workers’ turnover intention (M10, β = −0.422, *p* < 0.01), providing support for H1. This finding indicated that, in the gig economy context, algorithmic control was not merely perceived as an oppressive supervisory tool at the level of individual perception. When workers perceived that algorithms could provide them with resource support and an income guarantee, their turnover intention was significantly inhibited. From the perspective of Conservation of Resources Theory, algorithm systems provided workers with clear task structure, performance expectation and economic returns in different dimensions, and reduced the loss of cognitive resources caused by information asymmetry and role ambiguity. It should be noted that this conclusion only reflected correlations at the level of individual subjective perception, and did not deny the objective attribute of algorithmic control as a platform labor discipline mechanism. The asymmetric power between platforms and gig workers embedded behind it was the macro institutional background for the formation of individual perception.

#### 5.4.2. Mediation Effect Test

Second, hierarchical regression analyses in conjunction with the PROCESS macro were conducted to test H2 and H3, that is, the mediating effects of psychological contract breach and perceived usefulness. The results were presented in Table 4 and Table 5. Under the conditions of 5000 Bootstrap resamples, and a 95% confidence level, the indirect effect of perceived algorithmic control on turnover intention through psychological contract breach was −0.574, with a 95% confidence interval of [−0.782, −0.397]. Because the confidence interval did not include zero, the indirect effect was statistically significant, indicating that psychological contract breach significantly mediated the relationship between perceived algorithmic control and turnover intention. That is, perceived algorithmic control further reduced workers’ turnover intention by decreasing their psychological contract breach, thus supporting Hypothesis H2. Under the same conditions, the indirect effect of perceived algorithmic control on turnover intention through perceived usefulness was −0.327, with a 95% confidence interval of [−0.524, −0.168]. Again, the confidence interval excluded zero, indicating a significant indirect effect. This finding suggested that perceived usefulness significantly mediated the relationship between perceived algorithmic control and turnover intention. That is, perceived algorithmic control further reduced workers’ turnover intention by enhancing their perceived usefulness of algorithms, thus supporting Hypothesis H3. Taken together, both psychological contract breach and perceived usefulness exhibited significant mediating effects, providing support for H2 and H3. The establishment of the above mediating effects revealed dual mediation paths through which perceived algorithmic control influenced turnover intention. The first was the emotional and resource path based on contract fulfillment. This path was grounded in Conservation of Resources Theory and Psychological Contract Theory, and drew on the logic of labor process theory that algorithmic control restructured the implicit contractual relationship between platforms and workers. That is, algorithms reduced workers’ perception of psychological contract breach through transparent and fair rules, and then consolidated their psychological bonds with platforms to restrain turnover intention. The second was the cognitive and technical path based on instrumental value. This path took the Technology Acceptance Model as its core and explained the impact of the technical instrumental attribute of algorithms on individual perception and behavior. That is, algorithms enhanced workers’ perceived usefulness of algorithms by improving work efficiency and performance, thus forming positive attitudes toward the platform and further weakening turnover intention. The two paths jointly illustrated that the internal influence mechanism of perceived algorithmic control on turnover intention was the result of the dual resource protection process of individual emotion and cognition under the macro algorithm governance framework.

#### 5.4.3. Moderation Effect Test

To test the moderating effect of time pressure, perceived algorithmic control and time pressure were mean-centered prior to analysis, and their interaction term was constructed. Control variables, perceived algorithmic control, time pressure and the interaction term were sequentially included in the regression model. As shown in Table 3, the results of Model 4 indicated that the interaction term of perceived algorithmic control and time pressure had a significant negative effect on perceived usefulness (M4, β = −0.196, *p* < 0.01). This result suggested that time pressure negatively moderated the relationship between perceived algorithmic control and perceived usefulness; specifically, the positive effect of perceived algorithmic control on perceived usefulness weakened as time pressure increased. Accordingly, H5 is supported. This result indicated that time pressure changed workers’ cognitive processing of algorithmic control. Under low time pressure, workers had more cognitive resources to understand the logic of the algorithm, which made it easier for them to recognize its efficiency-enhancing functions and thus strengthened perceived usefulness. In contrast, under high time pressure, individuals’ cognitive resources were occupied by task execution, making them more likely to perceive the algorithm as a source of task pressure rather than an efficiency tool, thereby weakening its positive effect.

The results of Model 8 showed that the interaction term of perceived algorithmic control and time pressure had no significant effect on psychological contract breach (M8, β = 0.030, *p* > 0.05). This indicated that time pressure failed to significantly moderate the relationship between perceived algorithmic control and psychological contract breach, and Hypothesis H4 was not supported. The non-significant result of H4 might have been due to the fact that psychological contract breach reflected workers’ stable cognitive evaluation of the platform’s long-term fulfillment of obligations, which showed strong path dependence and accumulation. In contrast, time pressure was a short-term and situational stress state, and its fluctuation was unlikely to change workers’ fundamental judgment about whether the platform fulfilled its obligations in the short term.

**Figure 2:**
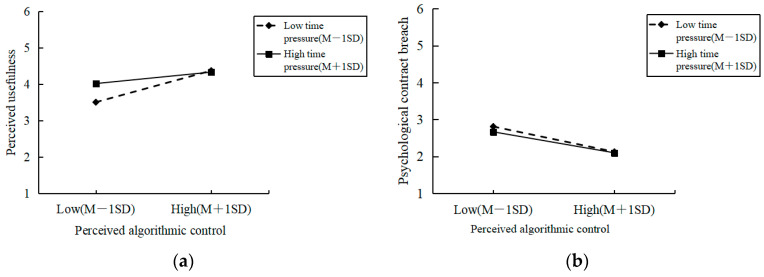
Moderating Effect. (**a**) Moderating effect of time pressure on the relationship between perceived algorithmic control and perceived usefulness; (**b**) Moderating effect of time pressure on the relationship between perceived algorithmic control and psychological contract breach.

To further examine the moderating pattern, simple slope analyses were conducted, and the interaction was plotted at high (mean + 1 SD) and low (mean − 1 SD) levels of time pressure. As illustrated in Figure 2, the positive relationship between perceived algorithmic control and perceived usefulness was stronger under low time pressure, whereas this positive relationship was substantially weaker under high time pressure. These results further corroborate the moderating effect proposed in H5.

#### 5.4.4. Moderated Mediation Effect Test

To further examine whether the mediating roles of perceived usefulness and psychological contract breach between perceived algorithmic control and turnover intention were significant under different levels of time pressure, this study conducted a moderated mediation effect test using the Bootstrap method, with the results presented in Table 6. When perceived usefulness served as the mediator, the indirect effect of perceived usefulness was −0.424, with a 95% confidence interval of [−0.687, −0.214] under low time pressure. Under high time pressure, the indirect effect of perceived usefulness was −0.154, with a 95% confidence interval of [−0.358, 0.002]. The index of moderated mediation was 0.129, with a 95% confidence interval of [0.018, 0.281]. Because this confidence interval did not include zero, the moderated mediation effect was statistically significant. This showed that time pressure weakened the mediating role of perceived usefulness between perceived algorithmic control and turnover intention. Accordingly, H7 was supported. When psychological contract breach served as the mediator, the mediating effect was significant at both high and low moderation levels. However, the index of moderated mediation was 0.044, with a 95% confidence interval of [−0.151, 0.247], which included zero. This indicated that time pressure failed to significantly moderate this mediating path, and Hypothesis H6 was not supported.

### 5.5. fsQCA Configuration Effect Analysis

The regression analyses conducted in the previous section identified the average relationships among variables and facilitated the interpretation of their overall patterns, whereas configurational analysis explored how different combinations of antecedent conditions influence the outcome variable. Combining these two approaches provided complementary analytical advantages, thereby offering a more comprehensive understanding of the interactions among variables and enhancing the explanatory power of the model.

First, turnover intention was treated as the outcome variable, and perceived algorithmic control, perceived usefulness, psychological contract breach, and time pressure were specified as antecedent conditions. Referring to existing practices in fsQCA research in the field of organizational behavior (Fiss, 2011), this study adopted the quantile calibration method for the data. Specifically, the 95th quantile was set as the full membership point, the 50th quantile as the crossover point, and the 5th quantile as the full non-membership point. This practice not only ensured the uniformity of calibration standards for each variable and the comparability of results, but also effectively adapted to the data distribution characteristics of the medium-sized sample in this study. In detail, using the 95th and 5th quantiles as anchor points effectively defined the boundaries of full membership and full non-membership of variable sets, thus avoiding the interference of sample extreme values on the judgment of conditional membership. The 50th quantile, as the crossover point, reflected the central position of the sample and was largely consistent with the middle level of the 5-point scale, helping to clearly distinguish the high and low set membership of variables. The results of the variable calibration are presented in Table 7.

Second, after completing the calibration process, a necessity analysis was conducted to examine whether any single antecedent condition constituted a necessary condition for the outcome variable. A condition was considered necessary when its consistency value exceeded 0.9. The test results were shown in Table 8. The results indicated that the consistency and coverage values of all antecedent conditions were below 0.9, suggesting that turnover intention arose from the joint effects of multiple factors, and no single antecedent condition alone could adequately explain the occurrence of gig workers’ turnover intention (Skaaning, 2011).

Finally, the fsQCA method was employed to identify the configurational pathways leading to turnover intention, with several analytical parameters specified during the analysis. The raw consistency threshold and case number were set to the software’s default values of 0.8 and 1, respectively, and the PRI threshold was set to 0.7 (Ragin, 2009). This study identified two main configurational paths, and this result reflected the high concentration of the formation mechanism of turnover intention to a certain extent. Specifically, psychological contract breach showed high consistency in the necessity analysis and thus became a core element of multiple paths in the configurational analysis. In addition, the research objects of this study were food delivery riders with high homogeneity, whose work situations, perceptions and understandings of algorithmic control, and resource acquisition modes were highly consistent, which reduced the space for the emergence of diverse configurational paths to a certain extent. Meanwhile, limited by the sample size, only a few configurations met the frequency and consistency criteria under a high consistency threshold, which might also lead to a relatively limited number of paths. Therefore, the results of this study reflected the stability of the core driving mechanism rather than a complex structure with coexisting multiple paths. The final results were shown in Table 9, with an overall consistency of 0.883 and an overall coverage of 0.746. The consistency of each individual path was greater than 0.85, indicating favorable configurational explanatory power.

Configuration 1 and Configuration 2 showed that high psychological contract breach was the core driving factor for the formation of gig workers’ turnover intention. On the one hand, low perceived usefulness meant that the algorithm failed to provide sufficient efficiency support, resulting in additional resource consumption, and psychological contract breach further disappointed the expectation of resource return. Dual resource loss significantly increased turnover intention. On the other hand, low perceived algorithmic control meant that workers believed the algorithm failed to provide sufficient task structure, feedback and resource support, while high psychological contract breach reflected that workers perceived the platform had not fulfilled its implicit promises. The combination of the two led to a decline in their trust and sense of belonging to the platform, which further triggered the turnover intention of gig workers.

To test the robustness of the configurational results, this study conducted a sensitivity analysis while keeping the frequency threshold unchanged. On the one hand, the consistency threshold was reduced from 0.80 to 0.75. On the other hand, the PRI threshold was raised from 0.70 to 0.75. The reanalyzed results showed that no substantial changes occurred in the configurational paths, their components, or core conditions under the adjusted parameters. Only slight fluctuations were observed in overall consistency and coverage, further supporting the robustness of the results.

## 6. Discussion

Before interpreting the following research findings, it is necessary to clarify the exploratory nature and contextual boundaries of this study. All conclusions are drawn based on questionnaire data from delivery riders on Chinese gig platforms, reflecting the psychological and behavioral patterns of this gig group under a specific cultural and institutional environment. Caution should be exercised when generalizing these findings to other gig work contexts. The relationships reported below focus on statistical correlations and underlying mechanisms among variables rather than causal relationships. The main findings of this study are as follows:

Perceived algorithmic control has a significant negative effect on gig workers’ turnover intention. The research results indicate that workers’ subjective perception still influences their behavioral responses to a certain extent, even under institutional arrangements with structural control characteristics. However, this finding does not mean that algorithmic control itself has supportive attributes, but rather reflects workers’ adaptive cognition and strategic responses under structural constraints. Therefore, the results of this study should be interpreted as revealing the cognitive and behavioral response mechanisms of gig workers under a specific algorithm governance context, rather than providing a simple tool for platforms to optimize worker retention and consolidate algorithmic control. Future research and management practices should go beyond focusing on the single indicator of turnover rate and place workers’ well-being, decent work, and sustainability in a more central position.

Further analysis of the underlying mechanisms reveals two pathways. On the one hand, perceived algorithmic control can reduce gig workers’ turnover intention by decreasing their level of psychological contract breach; on the other hand, it can also reduce turnover intention by enhancing gig workers’ perceived usefulness of the algorithm. This finding differs from critical views on perceived algorithmic control in early studies, which hold that perceived algorithmic control would inevitably increase workers’ sense of alienation (Wood et al., 2019). The findings support the predictions of the Conservation of Resources Theory. It shows that algorithmic control can reduce workers’ turnover intention by strengthening their sense of resource acquisition in the gig context. This echoes the argument proposed by relevant studies that algorithms act as empowering tools (Deng et al., 2025). It also expands the application boundary of this theory in the gig economy.

Time pressure plays a moderating role in the above mechanisms. Specifically, time pressure negatively moderates the effect of perceived algorithmic control on perceived usefulness and further weakens the mediating role of perceived usefulness in the relationship between perceived algorithmic control and turnover intention. However, time pressure does not significantly moderate the effect of perceived algorithmic control on psychological contract breach, and the moderated mediation effect is not supported either. This result may be explained by several factors. First, from the perspective of mechanism, psychological contract breach is essentially a relational cognitive evaluation formed on the basis of long-term interactive experience. Its core lies in individuals’ attribution judgment on whether the organization fulfilled its promises, rather than a direct response to the immediate work context. In contrast, time pressure mostly influences individuals’ immediate cognitive processing and task execution status. Thus, it more readily affects cognitive evaluation variables such as perceived usefulness, but is less likely to substantially alter individuals’ stable judgments of platform performance. Second, from the perspective of statistical testing, given the relatively limited sample size, the statistical power to detect interaction effects may be limited. As a result, existing moderation effects may be difficult to identify, but this does not necessarily imply that such mechanisms do not exist in theory. Finally, from the perspective of model setting, the influence of time pressure on individual cognition and behavior might not be a simple linear relationship. The linear test framework adopted in this study might fail to capture such patterns effectively. These alternative explanations do not deny the theoretical derivation of relevant hypotheses, but suggest that necessary caution should be maintained when interpreting non-significant findings. They also provide a new direction for future studies to explore other boundary conditions of psychological contract breach.

The configurational analysis results indicate that the formation of gig workers’ turnover intention is not the result of a single factor, but rather the joint outcome of multiple interacting conditions. High psychological contract breach emerges as the core driving condition leading to gig workers’ turnover intention, and its combination with low perceived usefulness or low perceived algorithmic control jointly contributes to the emergence of turnover intention. This configurational finding reveals that workers might still develop strong turnover intention under certain conditional combinations, indicating that platforms cannot rely solely on algorithmic control to retain workers.

### 6.1. Theoretical Implications

Specifically, the contributions of this study are as follows:

First, this study enriches the research on the impacts of perceived algorithmic control on gig workers’ behaviors. Existing studies primarily focus on the effects of perceived algorithmic control on workers’ occupational well-being, work behaviors, and work performance. However, research examining the relationship between perceived algorithmic control and turnover intention remains limited, and few studies examine workers’ stay-or-leave decisions in the highly fluid context of the gig economy. This study explores the mechanism through which perceived algorithmic control influences turnover intention through empirical analysis and constructs a dual mediation path of psychological contract breach and perceived usefulness. It reveals that algorithmic control is not merely a control mechanism, but also strengthens workers’ perceptions of resource acquisition and exchange fairness from both psychological and cognitive perspectives, thereby reducing turnover intention. This provides a new research perspective for alleviating the high mobility of workers in the gig economy.

Second, this study enriches the application context of psychological contract theory. This study finds through empirical analysis that psychological contract breach still plays a significant mediating role between perceived algorithmic control and turnover intention, even in the absence of formal employment relations. This finding indicates that the formation of psychological contract breach does not necessarily rely on legally defined employment relations, but can stem from workers’ subjective evaluation of whether the platform algorithm system fulfils its implicit promises. More importantly, this study reveals that the subject of psychological contract fulfillment may shift from specific organizational relations to abstract algorithmic systems in the gig economy. This transformation means that the antecedents of psychological contract breach expanded from previous interpersonal interactions to human–computer interaction, which represents an extension of psychological contract theory.

Finally, considering the unique working conditions of gig workers, this study incorporates time pressure as a moderating variable to examine its boundary effects on the relationship between perceived algorithmic control and turnover intention. Although time pressure exhibits a significant negative moderating effect in the perceived usefulness pathway, it does not exert a significant effect in the psychological contract breach pathway. The theoretical value of this finding lies in its exploration of the different sensitivities of the two mediating paths to situational pressure. As a cognitive evaluation based on immediate task performance, perceived usefulness is more susceptible to short-term time pressure. By contrast, psychological contract breach, as an emotional evaluation based on long-term mutual trust, is relatively stable. This suggests that future research can examine moderating variables based on the attributes of mediating variables, such as whether they are cognitive or affective, and short-term or long-term, when constructing moderation models. In addition, this study found that time pressure did not moderate the path of psychological contract breach as expected. This result is also theoretically meaningful, as it challenges the assumption that pressure necessarily amplifies all negative perceptions.

### 6.2. Managerial Implications

First, platforms can optimize algorithm system design. While ensuring efficiency, algorithms should be designed to be as transparent and flexible as possible. Meanwhile, platform enterprises should move beyond the misconception of designing algorithms with the primary goal of reducing turnover, and instead optimize algorithm systems with a focus on protecting gig workers’ legal rights and improving labor well-being. Through such optimization, workers’ perceived usefulness of algorithms can be effectively enhanced, and the risk of psychological contract breach can be reduced. This helps achieve a balance between platform development and the protection of workers’ labor rights, thereby improving workers’ well-being rather than merely maintaining organizational stability.

Second, this study finds that time pressure negatively moderates the effect of perceived algorithmic control on perceived usefulness. That is, high time pressure weakens workers’ evaluation of the perceived usefulness of the algorithm. This indicates that platforms should consider the negative impact of time pressure on workers’ cognition and attitudes, set reasonable time limits and take targeted measures to mitigate time pressure. For example, during peak hours or bad weather, platforms can appropriately relax the time limits for order receiving and delivery, establish a dynamic reward and compensation mechanism or provide an order receiving buffer period to help workers relieve the sense of time urgency. In addition, platforms can enhance workers’ time management and task coordination abilities through training, thereby improving their adaptability to and sense of control over algorithm tools.

Finally, platforms should establish effective communication, training, and fair incentive systems. First, platforms should establish regular communication channels and record problems and suggestions encountered by workers in a timely manner. When updating algorithms or adjusting policies, platforms should communicate with workers in advance, explain the reasons for and expected impacts of such changes, so as to avoid misunderstandings and trust issues caused by information opacity. Second, platforms should regularly conduct worker satisfaction surveys and set up worker representatives to participate in rule evaluation, so that workers can have a certain voice in algorithm management and enhance their sense of belonging and identity to the platform. By constructing a contractual relationship based on mutual trust and reciprocity, platforms can establish both emotional and institutional bonds in addition to technical control, improve workers’ perceived usefulness of the algorithm, reduce the risk of psychological contract breach, and ultimately restrain the emergence of turnover intention.

### 6.3. Limitations and Future Research

This study has certain limitations that need to be addressed in future research.

First, the sample of this study is mainly drawn from food delivery riders on Chinese gig platforms. Although this sample is somewhat representative in gig economy research, the relatively limited geographical scope and sample type might impose certain restrictions on the external validity of the research findings. For this reason, this study does not fully examine the heterogeneous role of social structural differences in the relationship between perceived algorithmic control and turnover intention. Existing studies show that gig labor is deeply shaped by social inequality structures and institutional contexts (Giuliani & Paraciani, 2025). Variations in resource endowments and institutional constraints across groups might lead to systematic differences in their perception of algorithmic control and behavioral responses. Specifically, female gig workers might be more sensitive to time pressure as they take on more family care responsibilities, which would further influence their turnover decisions. Workers with migrant backgrounds might rely more on platform jobs due to “ethnic penalties” in the labor market, and their evaluations of algorithmic control might also differ (Churchill et al., 2019). However, limited by cross-sectional data and sample conditions, this study does not systematically test the above heterogeneous mechanisms. Future research could extend to different countries and regions, and include various types of gig workers such as ride-hailing drivers and domestic workers, to further explore the boundary conditions and social stratification effects of perceived algorithmic control on turnover intention. In this way, a more comprehensive and inclusive research framework could be built, so as to improve the external validity and explanatory power of the research findings.

Second, this study adopts well-established questionnaire scales developed in previous studies to collect data in multiple phases, and uses different analytical methods to test for common method bias. Although common method bias is controlled through procedural control and statistical tests, all questionnaire data are self-reported by participants with certain subjectivity, so the risk of common method bias still exists. Future research could adopt longitudinal tracking designs, natural experiments and quasi-experimental designs, or combine objective data from platforms with survey data for analysis, so as to test the causal direction more rigorously and further enhance the reliability of the conclusions.

Third, although the model in this study considers the moderating role of time pressure, it does not sufficiently incorporate individual-level psychological traits and situational variables, such as workers’ self-efficacy, trust in algorithms, and occupational attachment. It also does not distinguish between different types or sources of time pressure on individual behavior. Future research could further distinguish between types of time pressure (challenge time pressure and hindrance time pressure) and sources (external and internal sources of time pressure), or introduce other situational variables, so as to more comprehensively reveal the boundary conditions and interaction mechanisms of the influence of algorithmic control on workers.

## Figures and Tables

**Table 1:** Confirmatory Factor Analysis.

Model	χ^2^	χ^2^/df	CFI	TLI	RMSEA	SRMR
Five-factor model (1, 2, 3, 4, 5)	469.363	1.495	0.928	0.919	0.051	0.064
Four-factor model (1, 2 + 3, 4, 5)	602.861	1.896	0.868	0.854	0.069	0.082
Three-factor model (1 + 2 + 3, 4, 5)	783.910	2.442	0.785	0.765	0.087	0.091
Two-factor model (1 + 2 + 3 + 4, 5)	1033.898	3.201	0.670	0.642	0.108	0.107
Single-factor model (1 + 2 + 3 + 4 + 5)	1119.100	3.453	0.631	0.601	0.114	0.113

Note: 1 = perceived algorithmic control; 2 = perceived usefulness; 3 = psychological contract breach; 4 = time pressure; 5 = turnover intention.

**Table 2:** Descriptive Statistics and Correlation Coefficients.

Variables	Mean	SD	1	2	3	4	5	6	7	8	9
1 Gender	0.62	0.49	1								
2 Age	2.25	0.96	0.103	1							
3 Educational level	2.72	0.72	−0.101	−0.043	1						
4 Occupational type	0.72	0.45	0.030	0.127	−0.038	1					
5 Perceived algorithmic control	4.21	0.51	0.136	0.267 **	−0.159 *	−0.081	1				
6 Psychological contract breach	2.39	0.87	−0.192 **	−0.157 *	−0.094	0.056	−0.425 **	-			
7 Perceived usefulness	4.04	0.61	0.092	0.123	−0.129	−0.057	0.547 **	−0.485 **	1		
8 Time pressure	3.48	1.05	0.028	−0.060	0.020	−0.195 **	0.080	−0.074	0.180 *	1	
9 Turnover intention	2.44	0.96	−0.131	−0.151 *	0.005	−0.034	−0.422 **	0.757 **	−0.450 **	−0.072	1

Note: ** *p* < 0.01; * *p* < 0.05.

**Table 3:** Hierarchical Regression Results.

Variables	Perceived Usefulness				Psychological Contract Breach				Turnover Intention	
	M1	M2	M3	M4	M5	M6	M7	M8	M9	M10
Gender	0.070	0.017	0.012	0.024	−0.190 **	−0.149 *	−0.148 *	−0.150 *	−0.118	−0.076
Age	0.121	−0.023	−0.015	0.000	−0.152 *	−0.040	−0.042	−0.045	−0.138	−0.026
Educational level	−0.119	−0.042	−0.046	−0.032	−0.117	−0.176 **	−0.175 **	−0.177 **	−0.013	−0.073
Occupational type	−0.079	−0.012	0.013	0.011	0.076	0.024	0.019	0.019	−0.014	−0.066
Perceived algorithmic control		0.543 ***	0.531 ***	0.480 ***		−0.419 ***	−0.417 ***	−0.409 *		−0.422 **
Time pressure			0.139 *	0.193 **			−0.032	−0.040		
Perceived algorithmic control × Time pressure				−0.196 **				0.030		
R^2^	0.041	0.301	0.320	0.353	0.075	0.231	0.232	0.233	0.037	0.194
F	1.993	15.879 ***	14.345 ***	14.184 ***	3.774 **	11.045 ***	9.204 ***	7.880 ***	1.756	8.834 ***

Note: *** *p* < 0.001; ** *p* < 0.01; * *p* < 0.05 (the same applies below).

**Table 4:** Results of Hierarchical Regression Test for Mediating Effects.

Variables	Psychological Contract Breach		Perceived Usefulness		Turnover Intention			
	A1	A2	B1	B2	C1	C2	C3	C4
Gender	−0.198 **	−0.149 *	0.070	0.017	−0.118	−0.076	0.032	−0.071
Age	−0.152 *	−0.040	0.121	−0.023	−0.138	−0.026	0.004	−0.033
Educational level	−0.117	−0.176 **	−0.119	−0.042	−0.013	−0.073	0.055	−0.086
Occupational type	0.076	0.024	−0.079	−0.012	−0.014	−0.066	−0.083	−0.07
Perceived algorithmic control		−0.419 ***		0.543 ***		−0.422 **	−0.118 *	−0.249 **
Psychological contract breach							0.724 **	
Perceived usefulness								−0.319 ***
R^2^	0.075	0.231	0.041	0.301	0.037	0.194	0.596	0.264
F	3.774 **	11.045 ***	1.993	15.879 ***	1.756	8.834 ***	45.06 ***	10.966 ***

Note: *** *p* < 0.001; ** *p* < 0.01; * *p* < 0.05.

**Table 5:** Results of Bootstrap Test for Mediating Effect.

Indirect Effect	Effect	SE	95% Confidence Interval
Perceived algorithmic control → Psychological contract breach→ Turnover intention	−0.574	0.097	[−0.782, −0.397]
Perceived algorithmic control → Perceived usefulness→ Turnover intention	−0.327	0.091	[−0.524, −0.168]

**Table 6:** Results of Moderated Mediation Effect Tests.

Mediating Variable	Moderator Variable	Effect	SE	95% Confidence Interval	Index	95% Confidence Interval
Perceived usefulness	Low time pressure (M − SD)	−0.424	0.120	[−0.687, −0.214]	0.129	[0.018, 0.281]
	High time pressure (M + SD)	−0.154	0.091	[−0.358, 0.002]		
Psychological contract breach	Low time pressure (M − SD)	−0.607	0.135	[−0.905, −0.371]	0.044	[−0.151, 0.247]
	High time pressure (M + SD)	−0.512	0.151	[−0.828, −0.234]		

**Table 7:** Variable Calibration.

Variables	Full Membership	Crossover Point	Full Non-Membership
Perceived algorithmic control	4.755	4.4	3.145
Perceived usefulness	4.8	4.2	2.8
Psychological contract breach	3.8	2.2	1.4
Time pressure	4.67	3.67	1.67
Turnover intention	4	2.25	1.25

**Table 8:** Necessary Condition Test.

Variables	Consistency	Coverage
Perceived algorithmic control	0.535	0.512
~Perceived algorithmic control	0.737	0.718
Perceived usefulness	0.520	0.478
~Perceived usefulness	0.736	0.749
Psychological contract breach	0.839	0.856
~Psychological contract breach	0.409	0.375
Time pressure	0.611	0.553
~Time pressure	0.609	0.630

Note: ~ Indicates logical NOT.

**Table 9:** Configurations of Antecedent Conditions for Turnover Intention.

Configuration	Consistency	Original Coverage	Unique Coverage
~Perceived usefulness × Psychological contract breach	0.905	0.660	0.092
~Perceived algorithmic control × Psychological contract breach	0.897	0.654	0.086
Overall consistency		0.883	
Overall coverage		0.746	

Note: ~ Indicates logical NOT.

## Data Availability

The raw data supporting the conclusions of this article will be made available by the corresponding author upon reasonable request.
